# Microscopic and Structural Studies of an Antimicrobial Polymer Film Modified with a Natural Filler Based on Triterpenoids

**DOI:** 10.3390/polym14061097

**Published:** 2022-03-09

**Authors:** Olga Fedotova, Dmitry Myalenko, Nataliya Pryanichnikova, Elena Yurova, Evgeniya Agarkova

**Affiliations:** All Russian Dairy Research Institute («VNIMI»), 115093 Moskow, Russia; o_fedotova@vnimi.org (O.F.); n_pryanichnikova@vnimi.org (N.P.); e_yurova@vnimi.org (E.Y.); e_agarkova@vnimi.org (E.A.)

**Keywords:** antimicrobial, modified packaging, microstructure, betulin visualization, triterpenoids, polyethylene film, optical microscopy

## Abstract

The aspects of component visualization of the antimicrobial triterpenoids (betulin) additive, both on the surface and in the bulk of the polymer, constituting food film packaging, are considered. This paper presents new knowledge about the morphology and surface structure of modified films using three independent methodological approaches: optical microscopy; a histological method adapted to packaging materials; and a method of attenuated total internal reflection (ATR) spectroscopy in the infrared region with Fourier transform. The use of these methods shows the betulin granules, individual or forming chains. To visualize the antimicrobial additive in the polymer bulk, a modified histological method adapted for film materials and attenuated total internal reflection (ATR) spectroscopy in the infrared region were used with Fourier transform using a Lumos Bruker microscope (Germany) (ATR crystal based on germanium). Sample sections were analyzed using Leica 818 blades at an angle of 45 degrees. The histological method consists of the study of a biological object thin section, in the transmitted light of a microscope, stained with contrast dyes to reveal its structures, and placed on a glass slide. In the method modified for the present study, instead of a biological one, a synthetic object was used, namely the developed film materials with the addition of natural organic origin. Individual granules are about 2 µm long; chains can be up to 10 µm long. The thickness of the granules ranged from 1 to 1.5 microns. It can be seen that the depth distribution of granules in the film from the inner surface to the outer one is rather uniform. Spectroscopic studies using the method of automatic ATR mapping in the region of 880 cm^−1^ made it possible to evaluate the distribution of an antimicrobial additive based on triterpenoids on the surface and in the polymer bulk.

## 1. Introduction

The safety issues of polymeric materials for dairy and food products have recently become increasingly important [1,2]. In this regard, there is a growing interest in creating packaging with functional properties [2,3,4]. This is especially promising for solid food products [2,5]. Giving functional properties to polymer films intended for food packaging will reduce the risks of recontamination during packaging, as well as preserve products throughout their entire life cycle [2,4]. 

One of the ways to improve the mechanical or physical characteristics and adapt to certain operating conditions of polymeric materials used as packaging materials is their modification—a transformation characterized by the new property’s appearance. This can be achieved by subjecting materials to mechanical or chemical treatment [6,7]. In the dairy and food industries, the modification of traditional polymeric materials of the polyolefin class, such as polyethylene, polypropylene, and its copolymers, is of the greatest interest. 

Modern engineering and technology open up wide possibilities for targeted action on polymers at different stages of their production, processing, or use to impart certain desired properties to them [6]. The following main methods of directional change in properties are distinguished: physical, chemical, and physicochemical [7].

Physical modification is suitable for many types of polymeric materials: it is based on the influence of physical factors, as a result of which structural-physical, chemical, and other transformations can occur in polymers [8,9].

There are three main tasks that can be solved with the help of physical modification: [10] targeted regulation of the physicochemical and technological properties of oligomers and polymers at the stage of synthesis and processing; accelerating the production processes and improving the technology for producing polymeric materials; and improving the physicochemical and other operational properties of polymeric materials and coatings.

This is achieved through the use of technology for modifying the polymer base with natural organic and inorganic components, which, in combination, impart a bacteriostatic or antimicrobial effect [11,12,13,14,15,16,17,18,19,20].

The use of antimicrobial packaging in the dairy and food industries will solve several main problems: to extend the shelf life of food products, while reducing food waste [21], and also to stabilize products in storage throughout their life cycle. Antimicrobial packaging can be made by adding antimicrobial agents such as chitosan, essential oil, and others to systems [22,23,24,25,26,27,28,29]. Various types of synthetic antimicrobial agents are described in the literature, such as metallic silver and copper nanoparticles [30], including their oxides [31], clay nanoparticles [32], etc. The type of antimicrobial agents chosen may differ depending on which application and packaging material is used.

The technology for modifying polymeric materials [1,10,20] used in this work is based on the principles of combining the base polymer in the melt (low-density polyethylene was used in the work) and a modifying additive of natural origin [23,24,25,26,27,28,29,30,31,32,33,34,35,36]. The application of this method is not always successful, since the existing modifying components are not thermally stable and may lose their inhibitory properties at high temperatures [11,14,37]. The creation of polymer films with antimicrobial properties using melt-combination technology is possible only if the melting temperature ranges of the modifying components and the base polymer are comparable [38,39,40,41,42,43,44,45].

The relevance of the research, in addition to the above, lies in the lack of scientifically substantiated data on the structure of the new modified film material and the distribution of betulin organic particles in the mass and the polyethylene near-surface layer.

The work has significant potential for further scientific development and practical application, which consists of a deeper study of the processes occurring during the contact of antimicrobial film packaging materials with food products in the dynamics of their storage.

The novelty of the research lies in the following:

- For the first time, variants of a modified low-density polyethylene (LDPE) film with a natural hydrophobic filler based on triterpenoids were obtained by melt matching with confirmed antimicrobial activity;
- New knowledge about the film surface morphology and the structure of its side section was obtained using methods not previously used in polymer research (histological);
- It has been determined that the betulin distribution on the surface and in the bulk of the polymer is uneven; however, it has an antimicrobial effect in the case of forced contamination of the film by 2–3 orders of magnitude, depending on the selected microorganisms.

## 2. Materials and Methods

**Figure 1:**
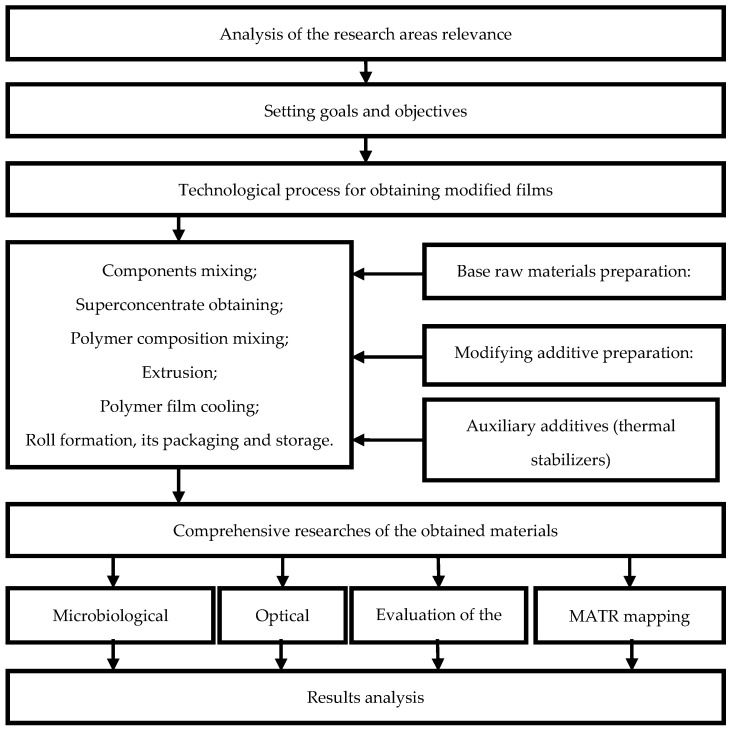
Schematic work diagram.

At the first stage of work planning, a research scheme was drawn up, as shown in Figure 1.

### 2.1. Research Objects

**Figure 2:**
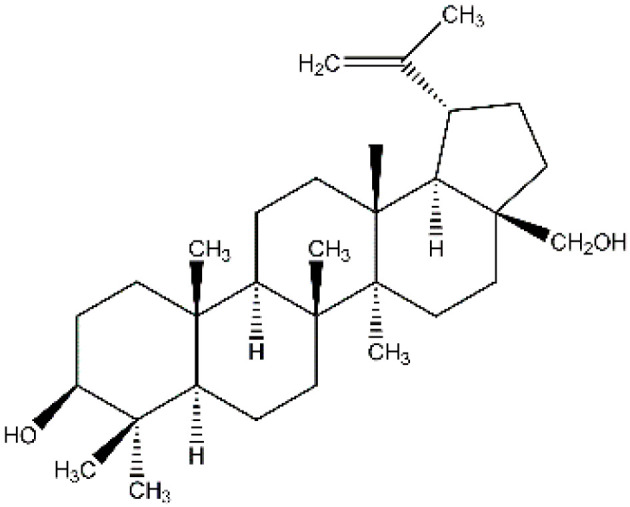
Betulinol molecule structure.

Birch bark extract was used as a modifying additive. It contains a number of triterpene pentocyclic compounds, consisting of betulin (betulinol), betulinic acid, lupeol, etc. [46,47,48]. The structural formula of the main extract active ingredient is shown in Figure 2.

Betulin, a naturally occurring triterpene, is usually obtained from the *Betula* L. birch bark [49]. The content of betulin and lupeol in the outer bark varies from 10% to 40% depending on the type of birch, its place and conditions of its growth, the age of the tree, and the season [50,51]. Betulin’s dependence on the birch type and variety is shown in Table 1 [52].

Betulin is a low-toxic compound (toxicity class 4): the half-lethal dose of betulin when taken orally is 9000 mg/kg. Betulin does not show allergenic, skin-irritating, sensitizing, carcinogenic, mutagenic, embryotoxic, or cumulative effects. The structure of the triterpenoid determines its affinity for biological membranes of human cells. Numerous biological studies indicate that betulin has high biological activity [53,54,55].

As a compound belonging to the class of triterpenoids, betulin has surface activity, exhibits the properties of an emulsifier and structure former. Betulin is slightly soluble in organic non-polar solvents (benzene, butanol) and fatty oils. The disadvantages of this compound include its low solubility in polar solvents (ethanol, isopropanol, water) [56].

Recently, research has been carried out to increase betulin solubility in various ways.

The inhibitory effect of this extract is given by betulin (C_36_H_60_O_3_), which is a triterpene alcohol, the concentration of which is at least 70.0% in the extract.

The used birch bark extract is approved for contact with food, is safe for humans, and has a neutral taste and smell. The literature sources describe in detail the betulin properties, its antibacterial, anti-inflammatory, and other functional properties [57,58,59].

The extract melting point is 258 °C (according to Gausmann) [41,47].

In practice, the process of obtaining various packaging materials with a selected extract can be implemented using extrusion or co-extrusion [1,43].

In this work, prototypes of polyethylene films (PE) based on low-density polyethylene (LDPE), the main physical and chemical properties of which are presented in Table 2, and an organic extract, the properties of which are given in Table 3, were obtained. To create a package with a given concentration of active betulin and its distribution over the volume of the polymer, the method of introducing a modifier through a superconcentrate was used [60,61,62]. It should be noted that this is a classical technique used to obtain films filled with various substances. Directly, the superconcentrate itself was made in several stages: preparation of the polymer base; preparation of modifier particles in certain concentrations; introducing the extract into the polymer matrix (melt mixing); and granule production (melting, cooling, cutting, and other technological operations).

To obtain a superconcentrate with the required characteristics, research was carried out on the effect of the concentration of betulin in the extract on the complex of properties of the resulting packaging materials [43,44,60,61].

The temperature regimes for processing the base of polyethylene are quite high (160–180 °C), and the extract itself is even higher (according to the manufacturer, the melting point of the obtained superconcentrate was 251–252 °C); therefore, in order to obtain high-quality prototypes of packaging, thermal stabilizers were introduced into the composition formulation. The process of obtaining a superconcentrate does not fundamentally differ from the traditional one. The obtained strands were cooled using a water chamber, with further cooling, granulation, and drying. In production, an extruder with 7 heating zones was used. Main technological characteristics of the superconcentrate: bulk density—0.48 g/cm^3^; melt flow index (MFI)—020, g/10 min; moisture content—no more than 0.10% [60,61].

**Figure 3:**
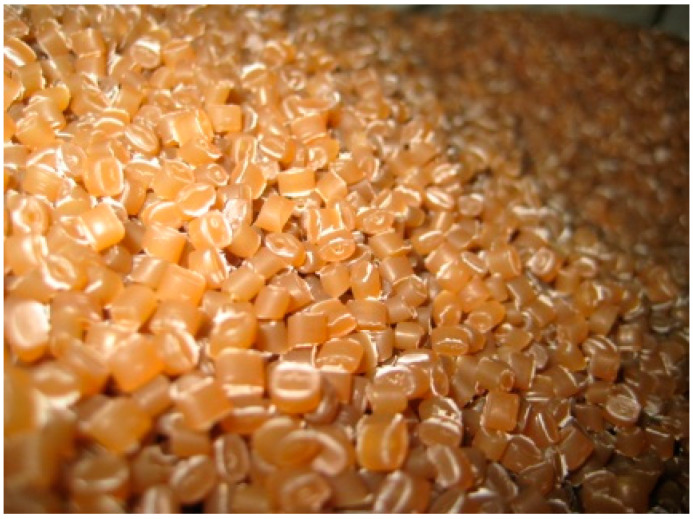
The exterior of the superconcentrate granules.

The exterior of the obtained granules resembles small cylinders, 2–5 mm in size and slightly yellow in color (Figure 3).

After obtaining prototype samples of the superconcentrate with different betulin content, research was carried out on the effect of its content on microorganisms located on the package surface. The concentrates presented in Table 4 were used in the tests.

From the literature data [43,44,61], it is known that betulin has high antimicrobial characteristics at an active substance concentration of at least 70%. In this work, to assess the microbiological parameters of the films, the range of betulin concentrations from 70% to 90% was chosen.

This range of concentrations was used in the first stage of the work to select the optimal one when creating a masterbatch on an LDPE matrix. At the second stage of the ongoing work, polymer films were obtained with a concentration of the antimicrobial component from 0.2 to 1.0 wt% in the finished product. Structural and microscopic tests were carried out.

On the basis of the above objects, plates with a flat surface were obtained by thermoforming. The size of the obtained samples was 100 mm long and 200 mm wide, with a thickness of 500 μm.

Polymer films were obtained on extrusion equipment using a ring die. LDPE brand 15803-020 was used as the base polymer. Processing was carried out under standard extrusion conditions without additional adjustment. The obtained film was cooled by air.

When evaluating the quality of the obtained samples, it was shown that the introduction of the extract does not affect the thickness variation of the finished polymer film. The film thickness in the sleeve was 45 ± 5 µm.

The production of multilayer polymer modified materials differs from the traditional processing of monofilms. Sample production was carried out on classical co-extrusion equipment without additional readjustment. Technical characteristics of the extrusion line were as follows: head Ø 190 mm; slot width 1.2 mm, and forming roll speed 10 m/min.

The resulting films had the following layer ratio: outer layer: polyamide (PA) 15 ± 3 µm; adhesive layer: 8 ± 2 µm; inner layer—LDPE 55 ± 5 µm. The conducted studies have shown that the resulting multilayer material has a uniform thickness at the level of 80 ± 5 μm. The introduction of the modifier does not lead to an increase in the thickness variation of the obtained samples. A visual assessment of the obtained samples showed the absence of visual defects.

### 2.2. Research Methods

#### 2.2.1. Optical Microscopy Methods

Visualization of the surface of the samples with different magnifications was carried out by the bright field method on an Axio Lab A1 transmission optical microscope with Axiocam 105 color optics at a final 1000× magnification.

Photographs of the sample surface of modified films containing various betulin amounts with 300× magnification were obtained using an Olympus BX50 microscope with an Ach 20×/0.40 Phz objective.

Two methods were used to visualize the antimicrobial additive in the polymer bulk:

(a) Histological method modified and adapted for film materials.

The principle of the method is to study a thin section of a biological object in the transmitted light of a microscope, stained with contrast dyes to reveal its structures, and placed on a glass slide [63]. In the method modified for the present research, instead of a biological one, a synthetic object was used, namely the developed film materials with the addition of natural organic origin.

Pieces of 15 × 15 × 4 mm^3^ in size were made from the film samples; at the same time, they were cut out at least three from different places of the film roll. The cut pieces were placed in a microtome, frozen to a temperature of minus (20 ± 3) °C, and sections were made, with a thickness of 10 to 30 µm. Sections were transferred under a light microscope.

The following analytical equipment was used in the study: stereoscopic microscope for transmitted light (Stemi 200—Carl Zeiss, Germany Oberkochen), microscope for transmitted light (Axio Imager. A1., Carl Zeiss, Germany Oberkochen), and Microm HM 525 cryomicrotome.

All obtained images were processed with the corresponding computer program ACD See Pro 9 in order to increase the possibilities of visual betulin differentiation inside the polymer film, on its surface, and during the sample’s storage.

It should be noted that this method was used for the first time to research objects made of synthetic polymers.

(b) Attenuated total internal reflection (ATR) spectroscopy in the infrared region with Fourier transform using a Lumos Bruker microscope (Germany Oberkochen) (germanium-based ATR crystal) [64,65]. 

Sample sections were analyzed using Leica 818 blades at an angle of 45 degrees.

The number of analyzed points for one sample is 50–70. Each material was subjected to spectral mapping in three planes (3 repetitions), while mapping was carried out perpendicular to the sample.

#### 2.2.2. Microbiological Research

To determine the inhibitory activity of the modifying additive containing betulin, we selected the following most representative surface spoilage microorganisms: *Escherichia coli* of CB), yeasts, and molds [40,44,60,66,67,68]. From the literature data, it is known that pure betulin has a consistently high inhibitory effect on selected groups of microorganisms [63,65].

One of the objectives of the research was to determine the effect of the extract concentration in the superconcentrate and, accordingly, in the obtained film packaging materials on their antimicrobial effectiveness, since the mixture of the polymer with the extract underwent significant thermomechanical effects during its processing.

The paper proposes a method of forced contamination of the surface of the obtained film materials with different extracts. The need for such a methodological approach is due to the fact that the determination of the microbial contamination of packaging objects is carried out by washing bacteria from surfaces [66]. This study required reliable knowledge of the number and species of surface spoilage microorganisms [61].

The inhibitory effect on the package surface was determined similarly to determining the effectiveness of aseptic treatment of the package surface by ultraviolet irradiation and was expressed as decimal logarithms of the number of microorganisms before the start of exposure (Nc) and after its completion (Nm) [69].

## 3. Results and Discussion

### 3.1. Visualization of Antimicrobial Additives on the Film Surface and in Polymer Mass by Optical Microscopy

With the naked eye, the presence of the additive in the obtained samples of packaging materials is not determined; therefore, it was of interest to conduct a series of studies on additive visualization in films.

**Figure 4:**
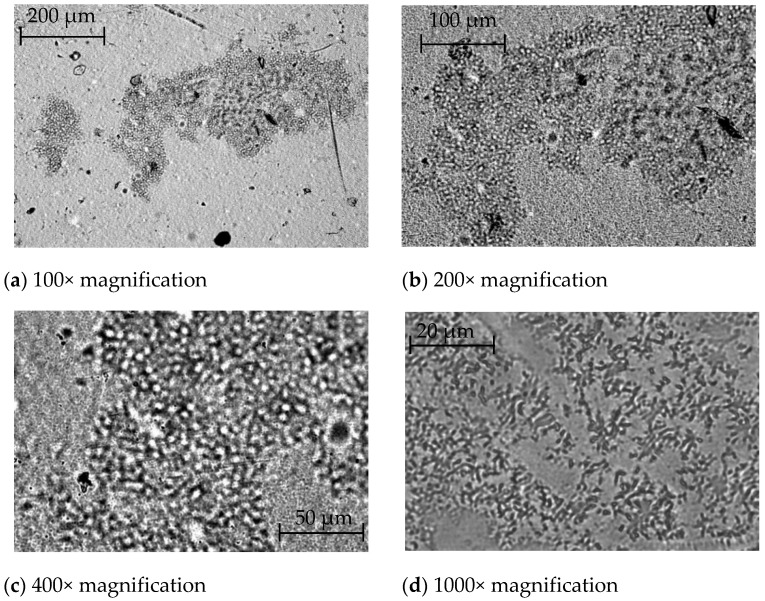
Microphotographs of the sample surface of LDPE film modified with betulin at different magnifications.

Visualization of the sample’s microstructure using the bright field method made it possible to obtain a more detailed picture of the surface structure of the modified LDPE film at different magnifications, as shown in Figure 4. In this case, magnification from 100× to 1000× was used [70].

Consideration of the obtained surface micrographs of the modified film showed that the introduced additive is visualized at all magnification options and that from 200× to 400× magnification is, in principle, sufficient to detect it in the polymer bulk [71].

Based on the obtained results, a simpler optical technique and 300× magnification were used to determine the change in the nature of the microstructure of the surface of modified films containing different amounts of the modifier.

**Figure 5:**
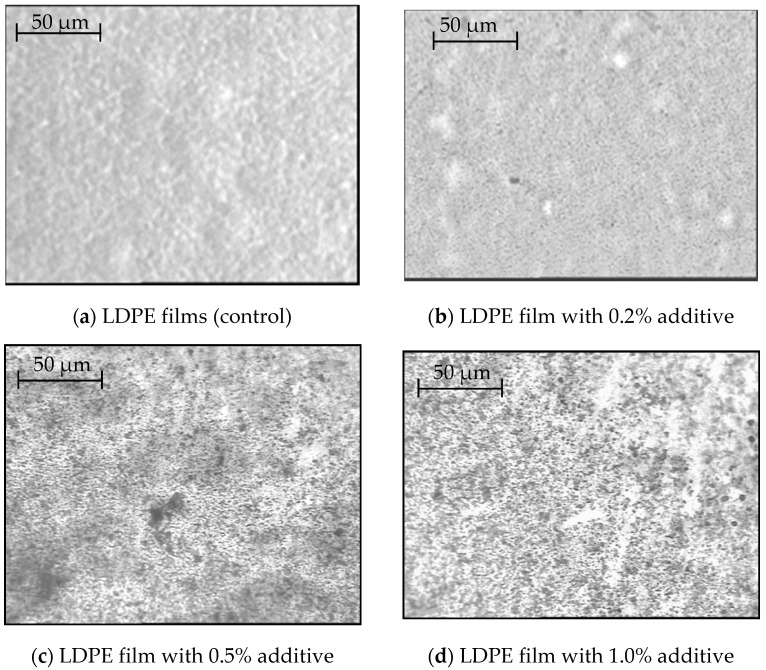
Photomicrographs of the surface of a betulin-modified LDPE film.

Figure 5 shows micrographs of film samples containing 0.2%, 0.5%, and 1.0% additives.

The presented photographs show particles of the modifying additive, the number of which increases with increasing concentration. Those, the microstructure of the surface of the sample’s changes with increasing concentration of the modifier.

**Figure 6:**
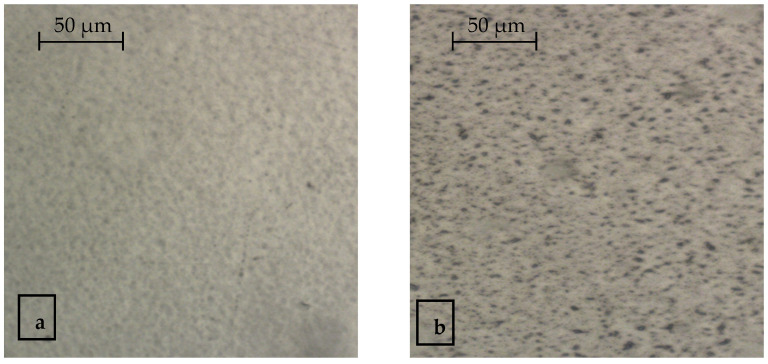
Surface microstructure of modified PA/LDPE film: (**a**) PA/LDPE (control), (**b**) PA/LDPE (0.5% additive in polyethylene layer).

A similar picture is observed when determining the surface microstructure of the resulting polyamide/polyethylene multilayer material, with a modified polyethylene layer (Figure 6).

Microphotographs of multilayer material samples are clearer when comparing the color range of the superconcentrate (Figure 1), and in the resulting picture, micro-inclusions of light-yellow color can be detected in the samples containing the additive (Figure 6b).

**Figure 7:**
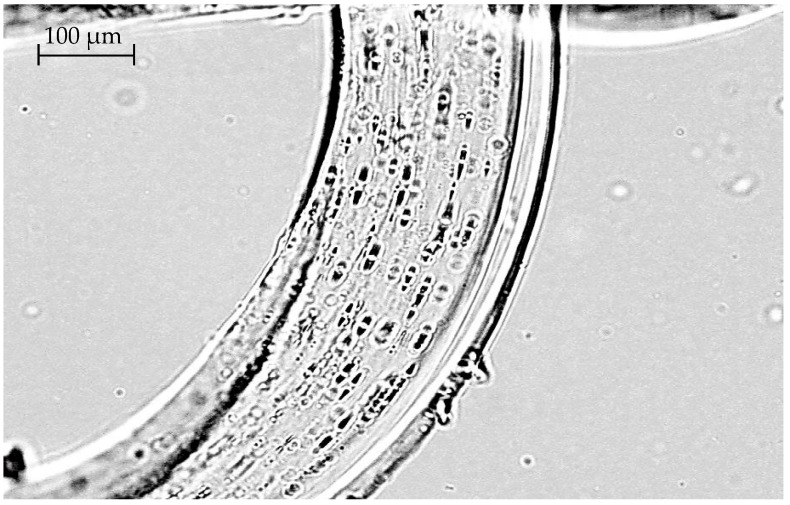
Cross section of a film with an extract concentration of 1% (63× magnification).

Figure 7 shows the results of a visual assessment of the presence and distribution of betulin inside the LDPE film. Film is cut across. The cut thickness is 15 microns. Betulin granules are visible, separate, or form chains. Individual granules are about 2 µm long; chains can be up to 10 µm long. The thickness of the granules ranged from 1 to 1.5 microns. It can be seen that the depth distribution of granules in the film from the inner surface to the outer one is rather uniform.

It was of interest to visualize the inner surface of the multilayer material using a modified histological method.

**Figure 8:**
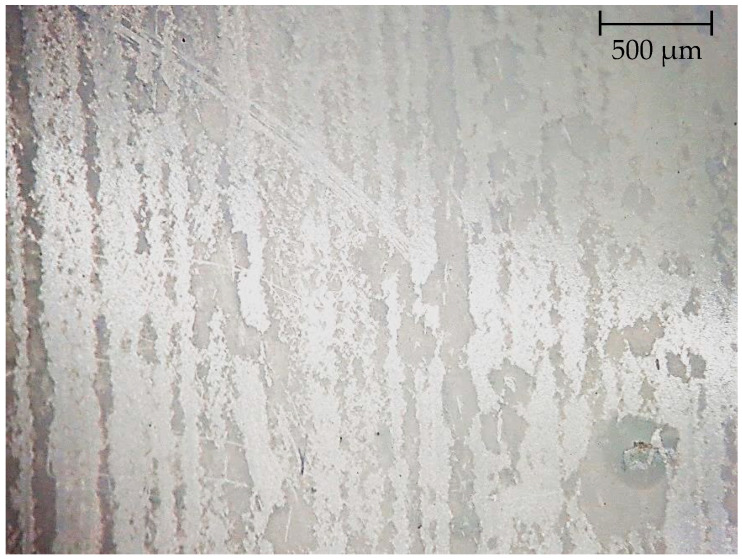
The inner surface of the film with betulin diffused from the film (2.3× magnification).

Figure 8 shows a microphotograph of the inner surface of a PA/LDPE laminate containing betulin.

Presumably, the diffusion of the additive takes place mainly in the zones of damage to the polymer integrity during its processing into a multilayer material.

Comparison of micrographs in Figure 6 and Figure 8 shows that the use of two different methods makes it possible to obtain a completely different picture of the surface microstructure. One unites them. In both micrographs, the polymer modifying additive is visualized; only in Figure 8, the structure of its orientation in the polymer mass was more obvious.

It follows from the presented data that, first, the method makes it possible to determine and visualize the presence of betulin both on the surface and in the polymer bulk, and second, the analysis of the cross section proves the presence of an additive in the bulk [60].

**Figure 9:**
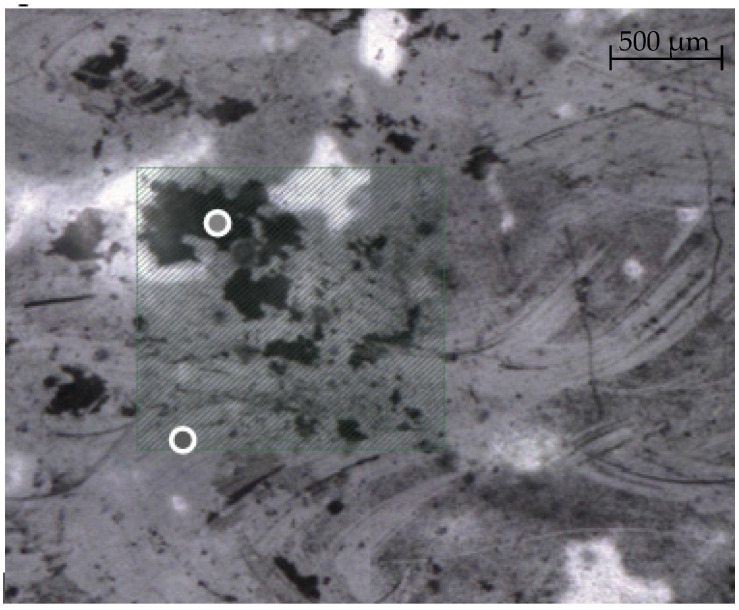
Internal Surface Mapping of PA/LDPE Multilayer Film.

Figure 9 shows the results of visualization of the surface of a multilayer PA/LDPE film from the side of modified polyethylene containing 0.5% extract.

**Figure 10:**
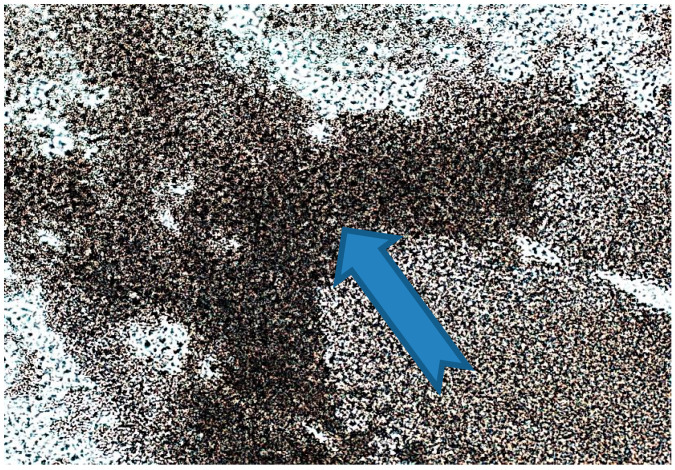
The inner surface of the “old” film with betulin diffused from the polymer is a dark zone. 20× magnification.

The square zone highlighted in Figure 10 is the automatic ATR mapping region. To determine the betulin distribution in the PE layer, automatic integration was performed under the peak in the region of 880 cm^−1^ [72].

It can be seen that in the researched region, there are both areas that do not contain an antimicrobial organic additive, as well as areas with increased content.

Comparison of visualization results in film material samples containing betulin showed that it is present both on the surface and in the mass of polyethylene and that its distribution is random.

### 3.2. Results of Microbiological Research

It has been established that the inhibitory ability of polymer packaging films with a natural antimicrobial betulin-containing additive in relation to sanitary indicative microorganisms (CB, yeast, and mold fungi) with an increase in the total amount of extract in the polymer mass by 2 times—from 10% to 20%—practically does not change. The efficiency in this range is only 3%.

The obtained experimental data on the effect of betulin concentration on the inhibitory ability of the film surface in relation to sanitary indicative microorganisms are presented in Table 5.

To determine the level of microbial contamination reduction of the film material samples’ surface, the antimicrobial efficiency index was used, which is expressed in Lg Nm/Nc, where Nc is the number of microorganisms on the surface of the control sample after exposure, CFU/cm^3^; Nm is the number of microorganisms on the surface of the modified sample after exposure, CFU/cm^3^ [60].

The results presented in the table are averages based on 5 parallel tests and show the high antimicrobial efficacy of the obtained film materials.

When more than 15% of the extract was added to the polymer mass, a noticeable foreign odor was observed in the obtained samples. This smell can be described as “bark smell”. The presence of extraneous stock is absolutely unacceptable for the packaging of dairy products. We chose the composition of a superconcentrate based on LDPE with the addition of 10% extract containing 80% betulin [60].

The manifestation of an inhibitory effect in prototypes of modified film packaging to selected microorganisms is hypothetically based on the principle of migration of antimicrobial components from the polymer mass to its surface, since the selected polymers mainly have an amorphous structure. The introduced organic and inorganic substances can be transferred from the surface of the packaging material to the product, while their concentration in the package gradually decreases, and in the surface layers of the product increases.

A preliminary economic calculation shows that the cost of obtaining a modified film with antimicrobial properties will be 1–5% higher than traditional polyethylene packaging, depending on the concentration of the introduced modifier. Industrial production of such packaging is possible on standard equipment without additional configuration and debugging, which also minimizes financial costs.

It should be noted that during the entire shelf life of the product, the concentration of the inhibitory natural component and the rate of its diffusion must be quite high in order to achieve the required level of bactericidal effect. This hypothesis was partially confirmed both by earlier studies [60] and by the fact that during long-term storage of samples, the additive practically “sweats” onto the surface, and this can be seen even with the naked eye. The observed effect is not observed in “fresh” samples. Figure 10 shows the picture of the mentioned “sweating” after 18 months of storage of samples, visualized using a modified histological method.

## 4. Conclusions

Thus, regularities in the distribution of the antimicrobial additive betulin in film materials were revealed by optical, histological, and spectroscopic methods.

It has been shown that when film and multilayer materials are obtained by combining the base polymer and the antimicrobial additive (betulin) in the melt, its distribution on the surface and in the bulk of the polymer is uneven and unsystematic. There are several ways to increase the uniformity of additive distribution in the polymer mass: the use of extrusion equipment with additional ultrasonic mixing, evaluation of the granulometric composition of the additive, or its sifting. At the same time, comparison of the results of these studies with the data on the antimicrobial activity of the developed materials shows that such a distribution does not affect the inhibition of surface spoilage microorganisms. The main factor affecting the antimicrobial activity is the concentration of the applied extract.

## Figures and Tables

**Table 1:** Betulin dependence on the birch type.

№	Birch	Betulin Mass Fraction, %
1	*Betula costata*	5
2	*Betula mandshurica*	27
3	*Betula pendula*	14
4	*Betula pubescens*	44

**Table 2:** Physical and chemical LDPE properties [6].

No.	Indicator	Dimension	Value
1	Density	g/cm^2^	0.918–0.930
2	Breaking stress in disease	kgf/cm^2^	100–170
3	Breaking stress in static bending	kgf/cm^2^	120–170
4	Breaking stress at shear	kgf/cm^2^	140–170
5	Relative strength at break	%	500–600
6	Elasticity modulus in bending	kgf/cm^2^	1200–2600
7	Hygiene yield strength	kgf/cm^2^	90–160
8	Relative attachment at the beginning of the current	%	15–20
9	Brinell hardness	kgf/mm^2^	1.4–2.5

**Table 3:** The main extract characteristics.

No.	Indicator	Actual Result
Physicochemical indicators *		
	Water mass fraction, %	Cream-colored powder, with a specific characteristic odor
2	Quantitative betulinol content, %	1.0
3	Water mass fraction, %	70–98
**Toxic elements**		
4	Lead, mg/kg	Less than 0.15
5	Arsenic, mg/kg	Less than 0.02
6	Cadmium, mg/kg	Less than 0.015
7	Mercury, mg/kg	Less than 0.01
**Pesticides**		
8	Hexachlorocyclohexane (α, β, γ-isomers), mg/kg	Not found
9	DDT and its metabolites, mg/kg	Not found
10	Heptachlor, mg/kg	Not found
11	Aldrin, mg/kg	Not found
**Microbiological indicators**		
12	QMAFAnM, CFU/g	Less than 10
13	CB (coliforms) in 0.1 g	NA
14	*E. coli* in 1.0 g	NA
15	Pathogenic, including salmonella in 10.0 g	NA
16	Yeast and mold, CFU/g	20

* Depending on the concentration of the initial active ingredient (betulinol), the physicochemical indicators of the additive may vary.

**Table 4:** Polyethylene-based concentrates.

Base Polymer	Polymer: Extract Ratio	Extract Content in Concentrate, %	Extract Concentration, %	Active Betulin Content, %
LDPE	9:1	10	70	4.90
LDPE	9:1	10	78	5.46
LDPE	9:1	20	78	5.46
LDPE	9:1	10	80	5.60
LDPE	9:1	10	90	6.30

**Table 5:** Efficiency of films disinfection with different content of betulin in relation to yeasts, mold fungi, and CB.

Additive Concentration (Betulin), %	Actual Result, Lg Nm/Nc		
	Yeasts	Mold Fungi	CB
70	1.00 ± 0.03	0.86 ± 0.04	1.00 ± 0.02
78	1.10 ± 0.04	0.96 ± 0.05	1.74 ± 0.02
80	1.18 ± 0.04	1.02 ± 0.04	1.76 ± 0.01
98	1.49 ± 0.05	1.00 ± 0.04	1.78 ± 0.01
